# Susac syndrome: neurological update (clinical features, long-term observational follow-up and management of sixteen patients)

**DOI:** 10.1007/s00415-023-11891-z

**Published:** 2023-08-22

**Authors:** Smriti Bose, Athanasios Papathanasiou, Sameep Karkhanis, Jason P. Appleton, Dominic King, Ruchika Batra, Susan P. Mollan, Saiju Jacob

**Affiliations:** 1 Department of Neurology, University Hospitals Birmingham NHS Foundation Trust, Birmingham, B15 2TH UK; 2 Department of Neurology, Nottingham University Hospitals NHS Trust, Nottingham, UK; 3 Institute of Applied Health Research, College of Dental and Medical Sciences, University of Birmingham, Birmingham, UK; 4 Department of Ophthalmology, University Hospitals Birmingham NHS Foundation Trust, Birmingham, UK; 5 Institute of Immunology and Immunotherapy, University of Birmingham, Birmingham, UK

**Keywords:** Susac syndrome, Encephalopathy, Sensorineural hearing loss, Branch retinal artery occlusion, Visual loss

## Abstract

Susac syndrome is a likely autoimmune microangiopathy affecting the brain, retina and inner ear. Due to the rarity of this condition, diagnosis and treatment can be challenging. Diagnosis is based on the presence of the clinical triad of central nervous system dysfunction, branch retinal artery occlusions and sensorineural hearing loss. Typical MRI findings of callosal and peri-callosal lesions may assist in diagnosis. Clinical course can be monophasic, polycyclic or chronic continuous. It is important to look out for red flags to attain an accurate diagnosis and follow a therapeutic algorithm based on severity of the disease and response to treatment. Patients are treated with steroids and immunosuppressive agents with a variable response. Early aggressive treatment especially in severe cases, may help in preventing relapses and morbidity/disability. This study highlights important diagnostic features and proposes a treatment algorithm based on clinical experience from management of 16 patients from 2 neuroscience centres in the UK since 2007, who were followed up over a long period of 3–15 years.

## Introduction

Susac Syndrome is a likely autoimmune condition consisting of a triad of encephalopathy, branch retinal artery occlusions (BRAO) and sensorineural hearing loss, first described in 1979 by Susac et al. [1]. Since then, 450 cases have been reported (case reports and a few case series) up until 2021 [2]. It is due to a microangiopathy affecting the precapillary arterioles of the brain, retina, and inner ear (cochlea and semicircular canals). The various components of the triad may present sequentially and often incompletely, leading to a delay in the diagnosis. Current understanding of this condition has been enhanced over recent years with more cases appearing in the literature and attempts to rationalise treatment strategies. Here we present an update based on our experience of managing patients, with an aim to shed further insight into the differences, challenges and vigilance required in the diagnosis and management.

## Methods

This study was a retrospective analysis of 16 patients diagnosed and managed across 2 tertiary level hospitals in the UK. All patients have been on regular follow-up since the time of diagnosis. Data was acquired from the institutional medical records.

The study was approved as a retrospective audit not requiring participant consent at the respective institutions (University Hospitals Birmingham Clinical Audit Registration and Management System, CARMS 13111, 08 November 2016 and CARMS 13667, 16 August 2017 and Nottingham University Hospitals, audit approval number 19-321Ca, completed on 9 December 2019).

## Results

### Demographics

Mean age of our patients was 35.6 years (SD = 10.1) ranging between 18 and 60 years. The female:male ratio was 3:1. Given the extreme rarity of diagnosis, we have consciously given the age at presentation in decades to avoid any inadvertent un-blinding of patients.

### Presenting complaints and investigations

Clinical and laboratory features are summarised in Tables 1 and 2. 75% of patients presented to the hospital with a subacute onset of headache and focal neurological deficits. More than half of the patients had either hearing or visual symptoms at onset. The complete clinical triad of symptoms involving the brain, eye and inner ear was seen only in 4 patients at presentation. Six more patients were found to have auditory or visual involvement either over time or after specialist investigations. 50% (8/16) of our patients fulfilled the definite and 43.75% (7/16) the probable diagnostic criteria [3] of Susac syndrome. Time from the onset of clinical symptoms to diagnosis ranged from 2 weeks to 10 months. A history of travel prior to onset of symptoms was present in a quarter of patients and a background history of mental health problems was noted in less than a third of patients.

**Table 1:** Clinical features, diagnostic tests, treatment and prognosis of sixteen patients with Susac syndrome

	Patient 1	Patient 2	Patient 3	Patient 4	Patient 5	Patient 6	Patient 7	Patient 8
Age (in decade)	31–40	41–50	31–40	41–50	31–40	21–30	21–30	11–20
Sex (M/F)	F	F	F	M	F	F	F	F
Presenting complaints	Three weeks intermittent headache with right hemi-paraesthesia preceded by scintillating spectra	Headache, persistent visual symptoms, memory problems	Six-week history of severe vertigo, headaches and vomiting	Vomiting, dizziness, confusion, headaches and ataxia following a viral prodromal illness	Five month history of episodic headaches, unsteadiness, episodic flashes, floaters and blurred vision, unilateral HL	Headache, facial numbness, confusion and diplopia	Intermittent episodes of unsteadiness, popping sensation in ears and blurred vision	Dizziness, forgetfulness
History of travel	Yes	Yes	Yes	Yes	No	No	No	No
Other associated medical conditions	Psoriasis, Chronic fatigue syndrome and anorexia	Chronic back pain, depression, epilepsy, diabetes and gastric banding	Depression and bipolar disorder	Rheumatoid Arthritis			IBS, cholelithiasis	Several infections in Dec 2016
Diagnosis at presentation	ADEM	Migraine	Probable Susac	Viral/autoimmune encephalitis and Marchiafava Bignami	Susac	Probable Susac	Susac	Probable Susac
Triad at presentation	No	No	No	No	Yes	No	Yes	No
Triad at Diagnosis	No	No	No	No	Yes	Yes	Yes	Yes
Time to diagnosis	8 months	12 months	1 month	12 months	5 months	1.5 months	3 months	1 month
CNS symptoms	Headache, encephalopathy, ataxia, dystonia	Headache, encephalopathy, focal limb and cranial nerve weakness	Headache, encephalopathy, dysphasia, memory loss	Headache, encephalopathy, focal signs, memory loss	Headache	Headache, encephalopathy, focal limb and cranial nerve weakness	Encephalopathy	Headache, Encephalopathy
Visual symptoms	Scintillating spectra	Persistent visual symptoms, diplopia	Nil	Nil	Flashes and floaters	Double vision	Visual loss	Nil
VA	Decreased	Decreased in left eye	Decreased	Could not be done as patient encephalopathic	Decreased	Normal	Normal	Normal
Fundus/FFA	FFA- no occlusions	FFA not done as fundus examination was normal	Blurred disc margins, microaneurysms, BRAO	FFA not done as fundus examination was normal	BRAO and venous occlusions	Not cooperative for FFA, OCT done	BRAO	BRAO
Vestibulo-cochlear symptoms	Hearing loss	Hearing loss, tinnitus	Hearing loss R, vertigo	Nil	Hearing loss	Nil	Hearing loss	Hearing loss
Audiogram	SNHL	Low frequency SNHL	Normal	Not done	R. SNHL	Low frequency HL	Low frequency HL	Mid frequency HL
MRI brain changes								
Callosal	Yes	Yes	Yes	Yes	Yes	Yes	Yes	Yes
Pericallosal	Yes	Yes	Yes	Yes	Yes	Yes		Yes
Infratentorial	Yes	Yes	Yes	Yes		Yes	Yes	
Enhancement			Yes	Yes	Yes	Yes	Yes	Yes
DWI restriction		Yes		Yes			Yes	
CSF protein g/L (normal range 0.15–0.45 g/L) OCB	1.35 Negative	0.26 Negative	1.02 Negative	1.1 Negative	No data	1.47 Negative	1.39 Negative	0.92 Negative
Other investigations	ANA 1:25, brain biopsy	RF, dsDNA	Positive serum EBV PCR	ANA 1:50, Anti GAD, (post-mortem brain histology)	EBV and CMV IgG			
Treatment	Steroids, IVIG, cochlear implant	Steroids, AZA, CYC	Steroids, CYC	Steroids, IVIG, PLEX, CYC	Steroids, IVIG, CYC, MMF	Steroids, IVIG, CYC, RTX	Steroids, IVIG, AZA, RTX, cochlear implant	Steroids, IVIG, MMF
Disease course	Relapsing—stable	Relapsing—stable	Monophasic	Relapsing—Death	Relapsing—stable	Monophasic	Relapsing—stable	Monophasic
Follow-up	13 years	3 years	8 years	1 year	5 years	5 years	7 years	4 years
Residual FNDs	Chronic Vertigo	Suicidal ideation, HL	Minimal cognitive deficits and occasional Vertigo		Full recovery	Walks with aid, low mood	Full recovery	Good recovery Occasional tinnitus

	Patient 9	Patient 10	Patient 11	Patient 12	Patient 13	Patient 14	Patient 15	Patient 16
Age (in decades)	31–40	31–40	31–40	31–40	31–40	31–40	51–60	41–50
Sex (M/F)	M	F	M	F	F	F	M	F
Presenting complaints at first presentation	Left visual loss	Headaches, sensory symptoms and bladder dysfunction	Headaches, encephalopathy, bilateral vision loss	Headache, encephalopathy	Headache, monocular field defect, tinnitus, unilateral hearing deficits, vertigo	Tinnitus, vertigo, hearing deficit, headache	Headache, tinnitus, right sided hearing loss	Tinnitus, right sided hearing loss
History of travel	No	No	No	No	No	No	No	No
Other associated medical conditions	Type 2 DM	Depression		Depression	Depression		Hypertension, Hypercholesterolemia	
Diagnosis at presentation	ON	MS	HSV	HSV	Susac	Susac	Probable Susac	Systemic vasculitis
Triad at presentation	No	No	No	No	Yes	Yes	No	No
Triad at final diagnosis	Yes	Yes	No	No	No	Yes	No	Yes
Time to diagnosis	8 months	4 months					1 year	7 months
CNS symptoms	Headache	Headache, L paraesthesia	Headache, encephalopathy, visual hallucinations	Headache, encephalopathy, gait apraxia	Headache,	Headache	Seizures, personality changes, cognitive deficits	Headache, Encephalopathy
Visual symptoms	L Visual loss	Nil	R blurred vision	Nil	Field defect	Nil	Nil	Nil
VA	Decreased	Normal	Decreased	Normal	Normal	Normal	Decreased	Normal
Fundus/FFA	BRAO	AWH	BRAO	AWH	Cotton wool spots	BRAO	Not done	Cotton wool spots
Vestibulo-cochlear symptoms	BL Hearing loss, tinnitus, vertigo	Hearing loss, tinnitus, vertigo	Nil	Nil	Hearing loss, tinnitus	BL hearing loss	Hearing loss, tinnitus	Hearing loss, tinnitus
Audiogram	Bilateral SNHL	Bilateral SNHL	Normal	Normal	Normal (not done at onset)	SNHL	Severe SNHL	Moderate to severe SNHL
MRI brain changes								
Callosal	Yes	Yes	Yes	Yes	Yes	Yes	Yes	Yes
Pericallosal	Yes	Yes	Yes	Yes	Yes		Yes	Yes
Infratentorial			Yes	Yes				Yes
Enhancement								
DWI restriction		Yes	Yes	Yes				
CSF protein g/L (normal range 0.15–0.45 g/L) OCB	0.724 Negative	0.436 Negative	3.3 Negative	0.8 Negative	0.48 Negative	Not performed Not done	0.744 Negative	5.0 Not done
Other investigations								Skin biopsy
Treatment	Steroids, MMF, aspirin, hearing aids	Steroids, AZA, aspirin	Steroids, aspirin	Steroids, aspirin	Steroids, aspirin	Steroids, aspirin	Clopidogrel	Steroids, CYC, aspirin, cyclosporin
Disease course	Monophasic (improved after higher dose of MMF)	Monophasic (improved after 1 year of AZA)	Monophasic	Monophasic	Monophasic	Monophasic	Monophasic (not offered steroids because of late presentation and patient choice)	Monophasic
Residual FNDs	Vision and hearing impairment	Depression, mild cognitive deficits, headaches, overactive bladder	Full recovery	Full recovery	Full recovery	Full recovery	Bilateral HL, mild cognitive deficit	Right sided HL

Raised CSF protein was defined as > 0.45 g/L, based on the laboratory range

*ADEM* acute disseminated encephalomyelitis, *ANA* anti-nuclear antibody, *AWH* arteriolar wall hyperintensity, *AZA* azathioprine, *BRAO* branched retinal artery occlusion, *CMV* cytomegalovirus, *CYC* cyclophosphamide, *DM* diabetes mellitus, *DWI* diffusion weighted imaging, *EBV* Epstein–Barr Virus, *FFA* fundus fluorescein angiogram, *FND* focal neurological deficits, *FU* follow-up, *GAD* glutamic acid decarboxylase, *HL* hearing loss, *HSV* Herpes Simplex Virus, *IBS* Irritable Bowel Syndrome, *IVIG* intravenous immunoglobulin, *MMF* mycophenolate mofetil, *MS* multiple sclerosis, *OCT* optical coherence tomography, *ON* optic neuritis, *RTX* rituximab, *SNHL* sensorineural hearing loss, *VA* visual acuity

**Table 2:** Summary of clinical features of sixteen patients with Susac syndrome

History of travel prior to onset of symptoms	4/16	25%
Presenting complaints		
Headache	12/16	75%
Eye symptoms	8/16	50%
Ear symptoms	9/16	56.25%
Confusion/forgetfulness	6/16	37.5%
Encephalopathy (at any stage)	11/16	68.75%
Psychiatric comorbidities	5/16	31.25%
CSF—raised protein	12/14	85.7%%
MRI		
Callosal lesions	16/16	100.0%
Pericallosal lesions	14/16	87.5%
Infratentorial lesions	9/16	56.25%
Enhancement	6/16	37.5%
DWI	6/16	37.5%
Hearing loss (at any stage)	12/16	75%
Audiometry abnormalities (at any stage)	11/14	78.6%
Objective evidence of retinal ischaemia, arterial occlusions on FFA/Fundus (at any stage)	11/16	68.75%
Monophasic course	11/16	68.75%

Encephalopathy was the most common central nervous system (CNS) manifestation after headache, seen at some point during the illness, although only 6/16 (37.5%) patients had it at onset. The ophthalmologic involvement was clinically silent and subtle on dilated retinal examination in some patients, in whom fundus fluorescein angiography (FFA) and optical coherence tomography (OCT) aided the diagnosis.

**Fig. 1:**
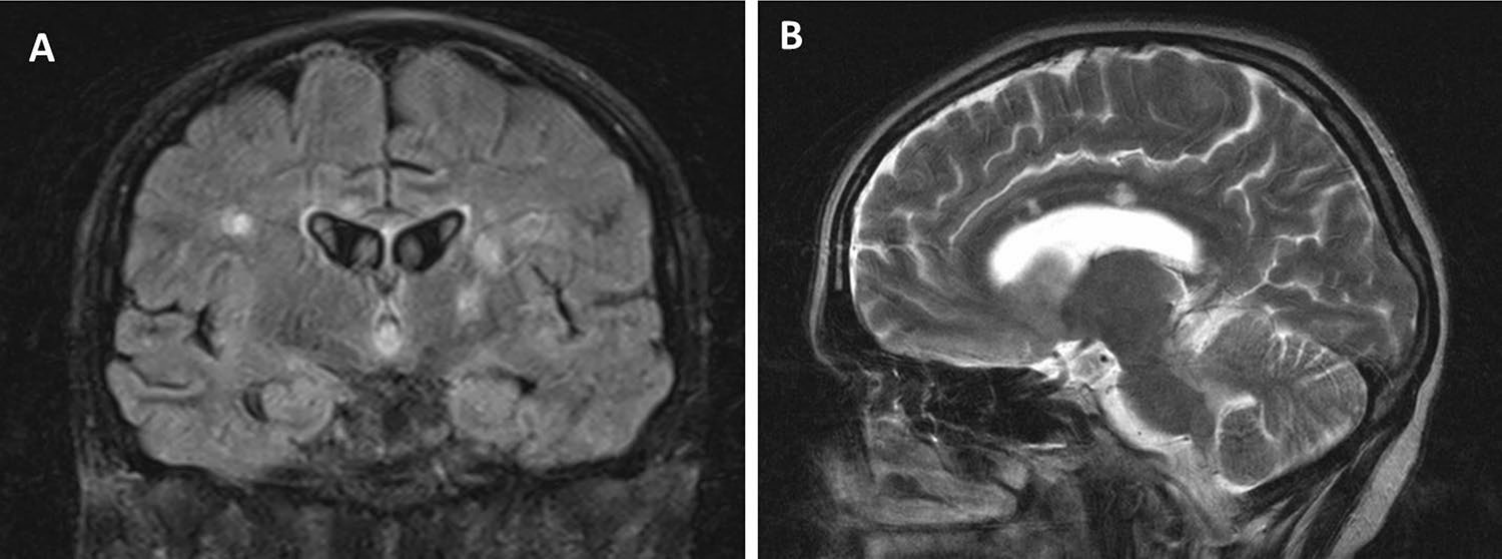
MRI changes in Susac syndrome MRI sequences of showing callosal (arrows), periventricular and thalamic lesions in coronal FLAIR (**A**) and sagittal T2W (**B**) images

Callosal and/or peri-callosal lesions on brain MRI were seen in all patients (Fig. 1). Only 1/14 patient had CSF leukocytosis (26 lymphocytes) and 12/14 patients had raised CSF protein. Oligo-clonal bands were absent in all 13 patients who were tested for it.

### Histological findings

**Fig. 2:**
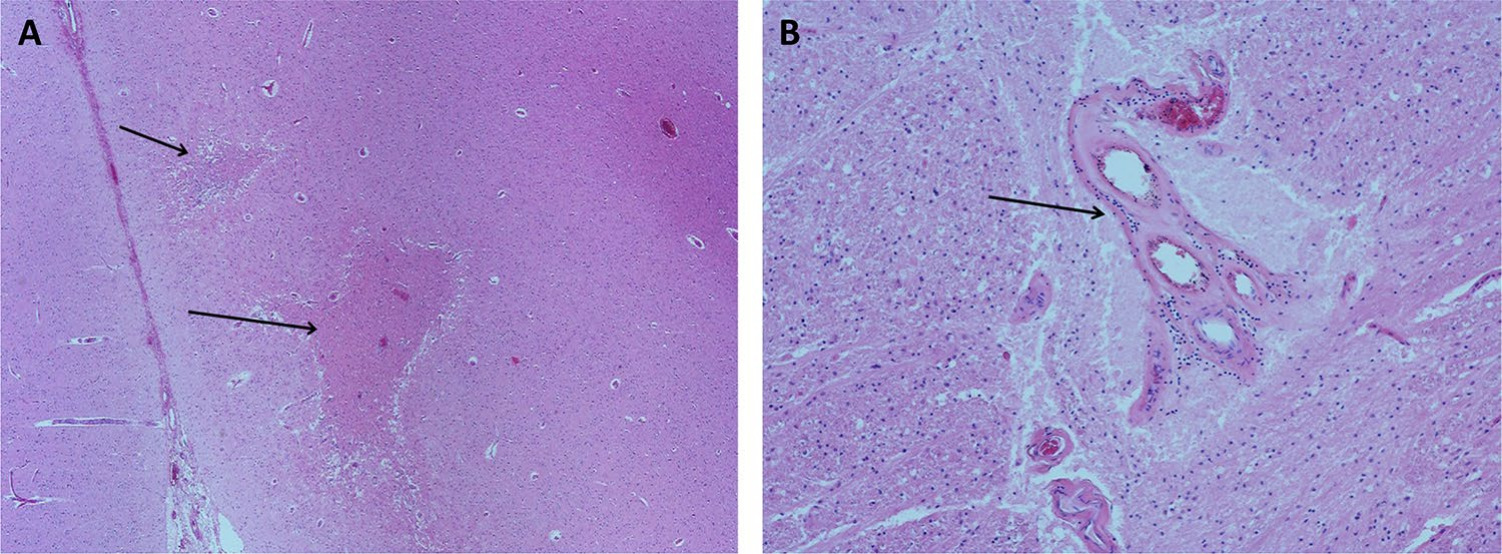
Pathological changes in Susac syndrome—brain histology showing multiple microinfarcts (arrows) in cerebral cortex (**A**, H&E × 40) with higher magnification (H&E, × 100) showing micro angiopathy with thickening of wall of arteriolar wall and sparse perivascular lymphocytic infiltrate (arrow) (**B**). *H&E* haematoxylin and eosin staining

One of our patients (patient 1) underwent stereotactic brain biopsy whilst another patient (patient 4) had an autopsy, both of which showed multiple microinfarcts involving grey/white matter, deep grey nuclei, brain stem and corpus callosum with endothelial cell necrosis and perivascular lymphocytic infiltration (Fig. 2A, B). C4d immunostaining showed complement deposition in the capillaries and venules in 30% of the vessels, suggesting humoral mediated microangiopathy. Patient 4 presented with encephalopathy and cognitive impairment with a compatible MRI but had no accompanying retinal or vestibulo-cochlear symptoms. Histology showed evidence of micro-infarcts and histopathological evidence of microangiopathy in the brain on postmortem study.

### Treatment

The various treatments used for our patients are demonstrated in Table 1. Of the 15/16 patients who were treated with steroids, 10 received Intravenous Methylprednisolone (IVMP) at presentation, and 3 others at some point during the course of illness. 2/16 patients received oral steroids at presentation. Intravenous immunoglobulin (IVIg) was more frequently used in relapsing disease course.

Not all patients were given long-term immunosuppression. All except 2 patients who had encephalopathy had a protracted course despite early treatment with IVMP. The relapses stopped after regular immunosuppression either with regular IVIG as in patient 1, or prolonged course of steroid-sparing agents (Patients 2, 3, 5, 6). Patients had fewer relapses on Cyclophosphamide and Mycophenolate mofetil as compared to Azathioprine. Interestingly, all patients who had Aspirin or Clopidogrel had a monophasic course (Table 1). Our experience allowed serial monitoring of some patients for over a decade (for up to 15 years) and despite best immunotherapy, several (> 50%) patients developed brain atrophy and cognitive impairment, very similar to what is often seen in other autoimmune central nervous system diseases like multiple sclerosis (MS).

**Table 3:** Medications used in management of Susac syndrome

Medication	Dosage	Summary of usage
IV methylprednisolone (IVMP)	1000 mg/day 3–7 days	All patients should have pulsed IVMP at onset for at least 3 days, with longer course for patients with more severe encephalopathic onset
Oral prednisolone	1 mg/kg/day (max 60 mg)—see titration suggestions	All patients should start oral Prednisolone at onset after the initial dose of intravenous steroids and maintained for at least 4 weeks, following which this can be reduced by 5 mg every 2 weeks until on 30 mg OD, then reduce by 5 mg every 4 weeks until on 15 mg OD, provided there are no relapses on dose reduction. Further tapering by 2 mg every month until on 9 mg OD and then by 1 mg every month and withdraw, especially if maintained on steroid-sparing drugs. In monophasic illness the dose can be tapered and stopped between 12 and 18 months
IV immunoglobulin (IVIg)	2 g/kg/day × 5 days	Our practice is to use IVIG in very severe encephalopathic onset or after first relapse. Many authors consider IVIG should be used as first line therapy along with corticosteroids
IV Cyclophosphamide (CYC)	10–15 mg/kg × three doses (2 weeks apart)	Unless major contraindications (eg: renal or concerns regarding ovarian failure), CYC can be used in refractory cases c. i. We generally prefer to maintain patients on other immunosuppressants (AZA/MMF etc.) in the longer term than oral cyclophosphamide maintenance to minimise side effects
Oral mycophenolate mofetil (MMF)	Start at 500 mg BD and increase to 1000 mg BD after 2–4 weeks	We find that MMF is better than other steroid-sparing immunosuppressants because of the faster onset of action needed to minimise longer term side effects. However, given the potential caution during pregnancy, this is probably avoided in young women when azathioprine or rituximab should be tried (If giving CYC, the lower dose can be started at onset with the dose increased to the maintenance level 2 weeks after the third cycle of CYC)
Oral azathioprine (AZA)	2.5 mg/kg/day	This was traditionally the first steroid-sparing drug used, although its slower onset of action and increasing availability of Rituximab has made this a less popular drug recently. However, this is still the drug of choice in young women because of the safety data in pregnancy
IV rituximab (RTX)	1 g/day × two doses (2 weeks apart)	Rituximab is now becoming an increasingly common drug especially if there is a relapse on any of the above treatments. CD19 levels can be monitored, and further doses given, if required
Oral aspirin (ASA)	75 mg/day	Although less commonly used, our experience has revealed that this is a safe drug

## Discussion and neurological update

### Epidemiology

Susac syndrome has a female preponderance with a female-to-male ratio of 2.2:1 and a mean age of onset of 30.5 years (± 9.6 years) [3] very similar to our observations. In a review by Dorr et al., mean age of presentation was 31.6 years with 81% falling between the ages of 16 and 40 years [4]. First presentation between ages 9 and 72 years was reported [5].

5% of the women had symptoms during or in the post-partum period [4]. Patient 12 in our series was 30 weeks pregnant at presentation. Cases have been reported from all continents. However, in a review of all reported cases, only 25% reported ethnicity, and 81% of these were white Caucasians [4].

### Pathophysiology

Autoimmune endotheliopathy seems to be the most likely pathologic process. One patient in our series had a probable clinical diagnosis of Susac, but had histopathological findings compatible with the diagnosis. Brain biopsies have shown micro-infarcts with microglial activation suggesting a T cellcell-mediated process [6]. CD8 T cell adherence to microvasculature (causing endothelial damage, vessel narrowing and occlusion) leading to microinfarcts was seen in transgenic mouse models and patients with Susac syndrome [7]. A recent study suggested similar pathological mechanisms between Susac, Rasmussens encephalitis and Narcolepsy type 1 [8]. Earlier reports supported the similarity to the pathogenesis of juvenile dermatomyositis where a different group of tissues were involved [9].

Neuropathologic studies are limited: findings reported so far include peri-arteriolar mononuclear infiltrate and collagen deposition, thickened arteriolar wall and basement membrane and microvascular fibrosis [10] [11], similar to what we have observed (Fig. 2). Petty et al. also found similar findings in the muscle of patients with Susac Syndrome [12].

Attempts at elucidating the pathophysiology of the condition were first made with brain biopsy in one of Dr Susac’s original patients, which demonstrated scarring representing a healed angiitis [1]. Other biopsies have found similar findings [13].

Preceding history of travel, insect bite, infection and serum Epstein–Barr virus (EBV)/cytomegalovirus (CMV) positivity in a few of our cases are pointers towards possible infectious triggers. Although not proven, the possibility of an infectious trigger has been proposed [14]. Petty et al., reported that 50% of patients in their study were CMV positive [12]. Four patients had low serum folate levels—the significance of this finding remains unestablished.

### Clinical features and diagnosis

All features may not be present at the initial presentation making the diagnosis challenging [3, 7, 9]. Clinical triad was fulfilled only in 13% patients at initial presentation and 80% of patients eventually, as reported in a review [4]. Although we had a higher percentage fulfilling the diagnostic criteria at onset, a lesser number of patients completed the triad on follow-up (Tables 1 and 2). Patients may present to ENT specialists and ophthalmologists at onset [15], who may not always be familiar with Susac Syndrome. The literature reports frequent misdiagnoses, often related to a lack of knowledge of this condition, both by physicians and radiologists, attributing MRI findings to multiple sclerosis or ADEM [16, 17].

Mean time to diagnosis was 3 months in our series, whereas it was 3 weeks in a case series of 7 patients [14] and up to 3 months in another study [15]. Diagnostic delay depends on the organs involved. A recent study reported a mean time to diagnosis of 3 months when the diagnostic criteria were met, 10 months with brain and retina involvement, 3 months with brain and inner ear involvement and 2.5 months with brain involvement alone [15].

Clinical features seen outside of the diagnostic clinical triad include skin manifestations like livedo reticularis and confusion [18], emotional disturbances, personality changes and psychiatric symptoms [19]. Studies are needed to evaluate the relevance of history of travel prior to onset of symptoms (Table 2) and a background history of mental health problems as seen 6/16 patients in our series (Table 1).

#### Brain symptoms

Encephalopathy and migraine-like headaches are the most common brain manifestations of Susac syndrome. However, encephalopathy may not always be present especially at disease onset [4, 7]. Presence of encephalopathy may mask the other symptoms involving the eye and ear (as seen in patient 7) and therefore a targeted search for their involvement is warranted in patients presenting with subacute onset of migraine-like headache and encephalopathy, especially in the presence of corpus callosal lesions.

CNS symptoms were the most common first clinical manifestation followed by an equal proportion of eye or ear symptoms. Migraine-like headaches may be present at onset in up to 80% of patients [4], quite similar to our cohort. Headaches have been associated with the imaging finding of leptomeningeal enhancement, suggesting meningeal inflammation as one of the possible causes for headache [20].

Neuropsychiatric presentations have also been observed, including behavioural changes and dementia [20] [21]. A background of mental health problems may therefore make diagnosis challenging especially in patients with a predominant encephalopathic onset.

#### Ear symptoms

Hearing loss may be the only presenting feature of Susac syndrome and it may be sudden or insidious in onset [5]. We found 9/16 of our patients who had hearing loss at onset. Sensorineural hearing loss, vertigo and tinnitus is most likely related to vestibulo-cochlear damage, and reports have mainly found audiometry deficits in the low to medium frequency ranges, averaging ~ 40 dB [22], as also seen in our series. Hearing loss tends to be progressive and bilateral, but asymmetric and higher frequency involvement may be due to involvement of larger aspects of the cochlea, suggesting more aggressive disease [23].

#### Visual symptoms

Retinal involvement may manifest clinically (visual loss, change in visual fields, scintillating scotoma, etc.) or be subacute and asymptomatic [23, 24]. The diagnoses in patients 5 and 6 were confirmed with the aid of specialist ophthalmology input. Only half of our cohort reported visual symptoms. A possible explanation could be the fact that retinal involvement is often peripheral and patients presenting with encephalopathy may not be able to accurately report visual symptoms. We would therefore recommend all patients with suspected Susac Syndrome to be screened by a senior ophthalmologist. The hallmark of the ophthalmological findings in Susac are FFA findings of BRAO as well as vessel wall hyper-fluorescence [4]. Endothelial damage may lead to extravasation of lipids and blood leading to formation of atheromatous plaques or Gass plaques. These, unlike true emboli (Hollenhorst plaques) which typically centre on the arterial bifurcation, are usually in the mid-arteriole [25]. The Gass Plaques are refractile and are best seen on clinical examination or fundus photography [23].

Two of our patients had arteriolar wall hyper-fluorescence as the only finding which would be clinically undetectable without FFA. This arteriolar wall hyper-fluorescence can be seen at the site of infarction but also at the site of vessel wall damage where infarction has not yet occurred [26]. Therefore, in those with encephalopathy and auditory findings suggestive of Susac Syndrome, without obvious retinal findings, FFA is essential to detect the early disease. This, however, might be difficult in an uncooperative encephalopathic patient.

FFA, like most other procedures is not without clinical risk [27]. However, the benefit of FFA is the early detection of retinal changes which will aid accurate and early clinical management, preventing visual loss, long-term disability and possibly long-term relapse.

OCT in acute stages may cause thickening of the retinal nerve fibre layer reflecting oedema from BRAOs. This may later resolve leading to focal thinning of the retinal nerve fibre and abnormal foveal contour. The findings differ from MS where a more diffuse involvement is seen (affecting the temporal nerve fibre layer and with normal foveal contour) [7].

Advanced ocular imaging modalities such as wide-field imaging, which as the name suggests, captures a wider retinal field, are useful for long-term retinal monitoring [28]. FFA may need to be repeated if no retinal abnormalities are detected because of the temporal disparity of the clinical symptoms and signs seen with the Susac triad and also because BRAO may recur over the course of illness [5]. OCT angiography for the screening of Susac Syndrome remains a research tool, due to the limited field of view [29].

Retinal micro-aneurysm is a new ocular finding in Susac syndrome which suggests ischaemic damage to the retina [30]. Two of our patients had cotton wool spots on fundus examination, which may also suggest the same pathology [3].

#### Radiology

Rennebohm et al. have suggested that the microinfarcts or “snowball” (larger lesions) appearance of the mid-corpus callosum and/or the “string of pearls” (micro-infarcts of the internal capsule) is perhaps sufficient to diagnose Susac Syndrome without evidence of hearing impairment or BRAOs [9, 31]. Although callosal lesions are included in the diagnostic criteria [3], they are not always present [32]; they are typically seen in the central fibres and splenium without involving the undersurface unlike MS [33]. The callosal roof is often involved giving an ‘icicle’ look to the lesions [7]. Callosal lesions are usually small and as a consequence of occlusion of the small (less than 100 µm) precapillary arterioles [33]. The periventricular white matter as well as the deep grey matter is also affected [7]; the deep grey matter can be involved in up to 70% of cases [5].

Leptomeningeal involvement can be seen in up to 33% [5] (Fig. 1). The correlation to headache however could not be established in our series. A plausible explanation is the variable course of the condition. Moreover, the lack of sensitivity of post-contrast T1-weighted images as compared to post-contrast fluid-attenuated inversion recovery (FLAIR) sequences may provide a reasonable explanation [34]. Spinal involvement has rarely been reported in Susac Syndrome [35, 36]. Small vessel or perivascular enhancement on high-resolution intracranial vessel wall MRI has been reported in Susac syndrome [37]. Degradation of fibres in the genu of corpus callosum, using Diffusion Tensor Imaging (DTI), is thought to be specific for Susac syndrome [38] and can potentially be a future diagnostic tool.

### Differential diagnosis

The prodrome of migraines, subacute onset and characteristic MRI lesions in the brain help differentiate Susac syndrome from remitting relapsing MS. The absence of oligo-clonal bands may be a useful marker to differentiate it from MS [4]. A common differential diagnosis at presentation is acute demyelinating encephalomyelitis (ADEM) and the presence of micro-infarcts (restriction on diffusion weighted imaging) may be beneficial in pointing towards a diagnosis of Susac syndrome in patients who do not meet the full diagnostic criteria. As Susac syndrome is a vasculopathy with clinical features similar to primary CNS vasculitis, it can often be misdiagnosed as the latter. MRI/CT angiogram and formal cerebral angiography can be beneficial in such patients.

### Clinical course

Clinical course was described as monophasic (fluctuating disease lasting less than 2 years), polyphasic or chronic continuous by Renneboum et al. [5], and in the 114 patients stratified in this manner 54% had monophasic, 42% had polyphasic and 4% had chronic continuous course [4].

A previous study suggested that clinical course may be self-limiting in those who develop encephalopathy within the first two years. MRI and FFA findings do not seem to be helpful in predicting the course of the illness [5]. There was no correlation between encephalopathy at onset and monophasic course in our series. In our experience, in terms of prognosis, those who had asymptomatic retinal findings of either arteriolar wall hyper-fluorescence or branch artery occlusions, tended to have a monophasic illness, which may indicate that early treatment has the potential to change the course of the disease by inducing remission. Conversely, those who reported photopsia symptoms, had a relapsing illness, which may indicate that these symptoms confer a worse prognosis. From our experience, in terms of prognosis, those who had asymptomatic retinal findings of either arteriolar wall hyper-fluorescence or branch artery occlusions and received appropriate immuno-suppression tended to have a monophasic illness.

Remissions have been noted spontaneously, [16] although patients can have a variable course and sometimes recurrence may occur after prolonged stability for several years [39].

A previous study found that most patients had a good recovery and brain atrophy seen on MRI may not always correlate with cognitive decline [40].

### Treatment

Randomised trials and prospective studies are lacking. Hence, all recommendations are from case series and expert opinions. Variable response to immunosuppressive treatment has been previously reported [5].

Most patients will require immunomodulation either in the form of steroids, IVIg ± steroid-sparing drugs. Only one patient from our cohort did not receive immunomodulation.

Moderate to severe encephalopathic patients may need more aggressive treatment (RTX or CYC) in comparison to those with only ear and/or eye involvement (MMF with or without MTX for 2 years after the relapse resolved). [9] Relapses have been reported during steroid taper and seem to improve with escalating the steroid dose [5].

A variety of treatment approaches have been tried to our patients (Table 1 and 2). The difference in therapeutic strategies reflects the variable response seen in this patient group and the variation to the severity of the disease. Other patients in the literature were treated in a similar fashion [3, 41]. Frequent Intravenous immunoglobulin (IVIg) may be of benefit in relapsing disease course.

We recommend early and aggressive therapy to prevent relapses because of the unpredictable course of the disease, and the potentially devastating neurological sequelae. There is limited experience of monoclonal antibody therapies for Susac, but some patients demonstrated remarkable improvement following their use [42]. The use of Rituximab in our series (patients 6, 7) was associated with a relatively good clinical response. There was complete return of visual acuity following treatment and patients remained relapse-free for a period of 4 years and 15 months, respectively. More studies on anti-endothelial cell antibodies may help justify treatment with B cell-targeted therapies [43]. Natalizumab in one report led to worsening symptoms [44], whereas off-label use in another study on 4 patients proved promising; however, the disease relapsed in 2/4 patients in the study when it was discontinued [45]. Infliximab may potentially be useful in refractory cases of Susac Syndrome [6].

We have changed our practice to include Aspirin in all new diagnoses over the last few years. Although anti-platelets were not officially included in the proposed treatment guidelines [9], they are commonly used in clinic practice [3, 46]. Their anti-inflammatory and antiplatelet properties may have a role to play. Longer-term prospective studies are needed to confirm or refute this observation.

**Fig. 3:**
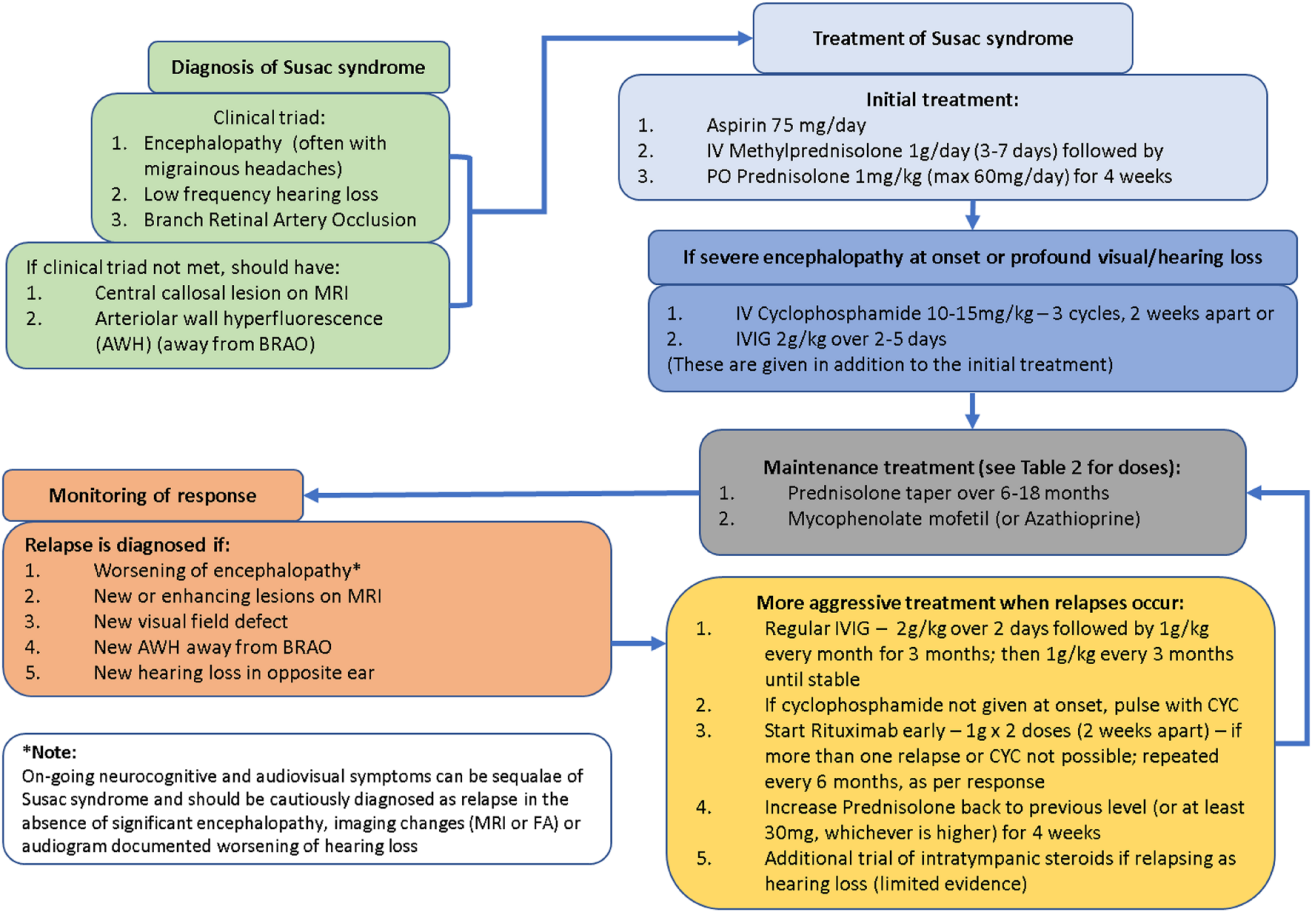
Proposed algorithm for managing Susac syndrome algorithm for diagnosis, treatment and monitoring of Susac Syndrome

Based on our experience, we have summarised the medical management of Susac Syndrome in Fig. 3 with the details of medications in Table 3. A consensus guideline for the management of Susac Syndrome has been recently published, which is not too dissimilar to our own recommendations [9]. Given the relapse potential of Susac Syndrome, strict follow-up is paramount [47].

### Organ-specific treatments (inner ear and retina)

Generally hearing impairment has been permanent with mild improvement at follow-up despite aggressive immunotherapy [48]. The higher susceptibility of the inner ear for irreversible damage due to ischaemia may account for lack of improvement.in hearing loss with treatment [7]. There are also previous reports of some improvement in hearing with intra-tympanic steroids [49]. Cochlear implantation in patients with hearing loss has been beneficial [50] as in 2 of our patients (patient 1 and 7). Retinal neovascularization may occur as a result of recurrent ischaemia [51] and photocoagulation, vascular endothelial growth factor inhibitor alone or together may be treatment options to prevent further complications [7].

## Conclusion

**Fig. 4:**
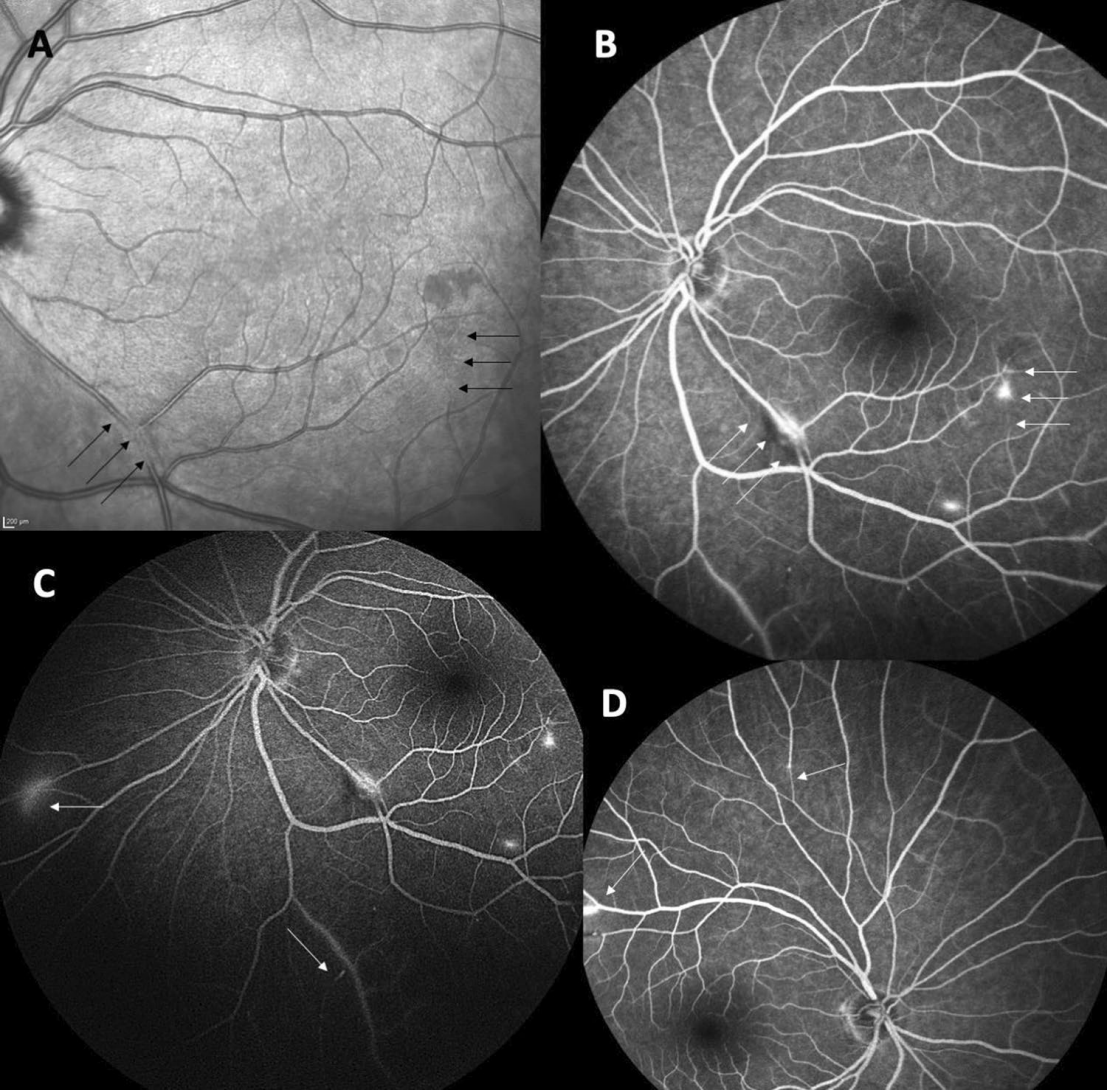
Fundal pictures in Susac syndrome optical coherence tomography (OCT) infrared image of the right eye (**A**) showing inferior retinal branch involvement (black arrows); the black arrows on the right of the image show occlusion, whereas on the left there is incomplete occlusion. Fundus fluorescein angiography (FFA), late phase of the left eye (**B**) in the same patient seen in A. There is arteriolar wall hyper-fluorescence seen along with frank leakage. FFA of the left eye (late phase, **C**) shows the requirement for peripheral views to gauge the extent of the retinal vascular involvement. FFA of the right eye (**D**, late phase) shows superior arterial tree involvement in the same patient

Susac Syndrome is a rare, under-recognised condition. Diagnosis is challenging as the complete clinical triad is not often seen at presentation, leading to misdiagnosis [3, 7]. Although the diagnostic criteria insist on the presence of symptoms suggesting vestibulo-cochlear impairment, patients presenting with encephalopathy may not be able to give a history suggestive of the same. Therefore, a detailed auditory and/or visual examination (including FFA and OCT) in suspected patients (e.g. migraine-like headaches, or/and encephalopathy with corpus callosum MRI lesions) is strongly recommended [3, 7] in order to assist in diagnosis. In our experience, FFA remains superior in terms of arterial wall hyper-fluorescence and evidence of fluorescence leakage distal to the wall hyper-fluorescence (Fig. 4A–D). In those with CNS symptoms and auditory compromise, a dilated ophthalmic examination, supported by wide-field retinal photography and OCT imaging should be performed [52]. Where there are CNS symptoms, no auditory compromise and where no retinal clinical signs are detected, the risk and the benefit of FFA should be discussed.

Early diagnosis and prompt treatment of patients with Susac Syndrome can result in resolution of symptoms, potentially curbing disease progression [3, 7]. In our experience, the disease severity, response to treatment and long-term prognosis are variable amongst patients, as previously described [3, 7, 9]. The mainstay of treatment is early prompt immunosuppression, as inadequate treatment might lead to irreversible damage [9]. On the other hand, there are cases (like our patients 11–14) that had a monophasic course with full recovery with steroids only. An individualised treatment approach based on severity at presentation [9] and response to first line immunosuppressive treatment is recommended based on our observations. Moreover, serial brain MRI imaging, OCT/FFA and audiometry for comparison is crucial in decision-making about acute and long-term immunosuppression.

Even though treatment is fundamentally by immunosuppression, optimal protocols and duration have not been clearly elucidated.

The role of RTX (or other similar B cell-targeted therapies) as a promising treatment in refractory patients hints towards the need for more studies evaluating its possible role in preventing relapses and progressive cerebral atrophy which may influence cognition in the long term.

The importance of starting Aspirin early-on to prevent micro-infarcts is also worth considering. Given the rarity of the disease, it is not surprising that randomised controlled trial evidence is lacking. The approach of utilising experience of treatment from other similar conditions is fundamental to current practice and allows anecdotal evidence and expert opinion to guide management. Even though the retrospective nature of the study is a limitation, the highlights of our study are the large cohort of patients with long-term follow-up. This patient group was diverse; the youngest patient was in the late teens and the oldest was in their 6th decade at presentation. Two patients had successful pregnancies post diagnosis. One patient had an aggressive disease leading to death, whereas 6 patients had a monophasic course not requiring long-term immunosuppression.

## Abbreviations

ADEM: Acute disseminated encephalomyelitis
AWH: Arteriolar wall hyperfluorescence
AZA: Azathioprine
BRAO: Branch retinal artery occlusions
CMV: Cytomegalovirus
CNS: Central nervous system
CSF: Cerebrospinal fluid
CYC: Cyclophosphamide
FFA: Fundus fluorescein angiography
H&E: Haematoxylin and eosin
HSV: Herpes simplex virus
IVIg: Intravenous immunoglobulin
IVMP: Intravenous methylprednisolone
MRI: Magnetic resonance imaging
MS: Multiple sclerosis
OCT: Optical coherence tomography
PCR: Polymerase chain reaction
PLEX: Plasma exchange
RTX: Rituximab
